# B Cells: The Old New Players in Reproductive Immunology

**DOI:** 10.3389/fimmu.2014.00285

**Published:** 2014-06-23

**Authors:** Franziska Fettke, Anne Schumacher, Serban-Dan Costa, Ana Claudia Zenclussen

**Affiliations:** ^1^Department of Experimental Obstetrics and Gynecology, Medical Faculty, Otto-von-Guericke University, Magdeburg, Germany; ^2^University Women’s Clinic, Otto-von-Guericke University, Magdeburg, Germany

**Keywords:** pregnancy, B cells, autoantibodies, IL-10, Breg, B10 cells

## Abstract

Reproductive immunology research has long focused on T cell responses to paternal antigens and tolerance mechanisms supporting fetal well-being. The participation of B cells herein was not widely studied. Because of the fascinating immunological uniqueness of pregnancy, it is however to be expected that such pleiotropic cells play a considerable role. In fact, on the one hand B cells contribute toward pregnancy tolerance by secreting the immunomodulatory cytokine IL-10 but on the other hand can seriously harm pregnancy because of their capacity of producing autoantibodies. As for protective B cells, new evidences in mouse models arise suggesting that IL-10 producing B cells, the so-called B10 cells, help in maintaining tolerance toward semi-allogenic fetal antigens. They may be also important to fight danger signals at the fetal-maternal interface as, e.g., in the case of infections with the aim to restore the disrupted fetal tolerance. In human pregnancies, IL-10 producing B cells increase with pregnancy onset but not in the case of spontaneous abortions. *In vitro*, they are able to suppress TNF-α production by T cells from pregnant individuals. Their generation and functionality will be discussed throughout this review article. B cells can be deleterious to pregnancy as well. Aberrant B cell compartment is associated with obstetric pathologies. In particular, the capacity of B2 cells to produce specific autoantibodies or of B-1a B cells to secrete natural autoantibodies that can turn autoreactive will be discussed herein.

## Introduction

The study of the mechanisms responsible for the paradoxical survival of the conceptus as an intra-uterine semi-allograft within the genetically distinct female host has been an area of substantial scientific devotion. Aspects, particularly those related to the role of the maternal adaptive and innate immune response at a time when the physiological unit of fetus and placenta is framed as well as fertility problems, recurrent miscarriages, premature deliveries, and pre-eclampsia have been widely studied.

B cells are a major component of the immune system thus likely to be involved in maternal fetal immune tolerance. This review will look back in time to the beginnings of B cell discovery, their functional diverse subpopulations and provide the reader with an au courant knowledge regarding their regulatory and pathogenic role in pregnancy. Genuinely B cells were not identified as cells but through their function of secreting antibodies. In the late 1890s, Emil von Behring and Baron Kitasato Shibasaburo described the appearance of protective antibodies in blood in response to introducing foreign antigens into the body. Kitasato and Behring demonstrated the value of antitoxin against diphtheria and tetanus toxins by means of transferring graded injections of blood serum from an animal infected with the disease to a non-immune animal, thus transmitting active humoral immunity and preventing the disease (1). However which cell types were involved in the generation of such antibodies was not discovered for another half century. Murphy and Morton documented lymphocyte infiltration in immunized mice during the process of rejecting inoculated cancer grafts, with either natural or induced immunity. Per contra the destruction of lymphocytes with repeated small doses of x-ray prior to introducing the cancer graft led to a loss of natural or induced resistance toward inoculated cancer growth and the tumor graft grew more readily (2). Following on Jerne postulated that an antigen binds to an antibody by coincidence and upon binding further antibodies to that antigen could be produced. Thus, this theory offered an explanation for the presence of natural antibodies (3); based on this Burnet published the theory of clonal selection in 1957. Herein, he proposed that each lymphocyte carries specific immunoglobulins on its cell surface, reflecting its specificity of the antibody that will be synthesized upon antigen stimulation (4). Nossal and Lederberg confirmed this hypothesis during the following year (5). In 1956, B cells were first identified in chicken by Glick and Chang. Their data demonstrated that the resection of the bursa of Fabricius, called bursectomy, led to a suppressed antibody response and in point of fact B cells did receive their name from this bursa, the place of B cell origin in young birds, and not the term bone marrow (BM) as one would believe because of their origin in humans (6). During the last decades, the key discoveries included the unravelment of the immunoglobulin structure, the antibody–antigen interaction, and the mechanisms involved in the generation of antibody diversity. In particular, the knowledge about the immunoglobulin structure triggered the development of synthetic derived monoclonal antibodies. A breakthrough in the field of specific gene modification in animals came with the isolation of embryonic stem cells and the discovery of homologous recombination (7, 8). Since then experimental models have undergone significant technological development, beginning with inbred mouse lines toward transgenic mouse models allowing for B cell stock manipulation in the 1980s and gene knockout animals in the 1990s (9).

## A Display of B Cells and B Cell Subsets

B lymphocytes are cells in the humoral immunity of the acquired immune system and account for 5–15% of circulating lymphocytes (10). Today, they are classically defined via the presence of endogenous immunoglobulins. A common description is that of a cell population expressing “clonally diverse cell surface immunoglobulin receptors,” which recognize specific, antigenic epitopes (11). In adult human subjects, as in all mammals, B lymphocytes are continually formed in the BM from committed pluripotent hematopoietic precursor cells (12). Preceding the BM is populated by hematopoietic stem cells originating from the fetal liver (13). The earliest committed precursor of the B cell lineage is the pro-B cell. Downstream, functionally immature B cells also known as naive B cells (co-expressing IgM and IgD) exit the BM and migrate to the spleen, where they differentiate through transitional stages into B1 cells (albeit not all B1 cells derive from the BM) follicular B cells or marginal zone (MZ) B cells. B1 cells, independently of their origin, are typically subdivided into B-1a and B-1b cells (14).

The principal function of B lymphocytes is the production of antibodies against microbial antigens. Naive B cells not yet exposed to an antigen habitually recirculate secondary lymphoid tissues, chiefly spleen and lymph node follicles in order to encounter antigens. B cell activation, explicitly proliferation and differentiation, is mediated through positive and negative regulation in gene expression upon antigen encounter. Eventually, most B cells differentiate into antibody-secreting plasma cells while a small minority persists as memory cells, the agent of lasting immunity. Crucially, while first exposure to an antigen results in the generation of IgM secreting plasma cells and memory B cells (primary immune response), repeated activation of these memory cells by the same antigen leads to the production of a large quantity of high-affinity, monospecific class-switched IgG antibodies (secondary immune response). The differences of these antibody categories and their role in autoimmunity and pregnancy will be considered.

Beyond the widely recognized role of B lymphocytes in antibody production, B cells can also act as antigen presenting cells (APCs) for the initiation of T cell immune responses, as demonstrated in B cell depleted mice (15). B cells act as APCs by the presence of a transmembrane receptor protein on their surface known as the B cell receptor (BCR). Additionally, B cells can regulate various T cell and DC functions through the secretion of immunomodulatory cytokines (16–18). A novel but less well understood concept describes a phenotypically distinct subset of regulatory B cells to negatively regulate cellular immune responses and inhibiting excessive, tissue specific inflammation (19).

Like other cells, B lymphocytes can be classified into subsets according to their variation in development, anatomical location and ability to migrate, surface marker expression and functional characteristics. Their discovery has been facilitated by means of phenotype recognition using multicolor flow cytometry (20, 21). Two major B cell populations have been described; namely B1 cells and B2 cells.

Follicular B cells and marginal zone B cells (MZ B cells) constitute the B2 cell population. Together, they make up the chief part of splenic B cells but differ among each other in anatomical location, the follicles, and MZ, respectively. Follicular B cells are produced postnatally from BM precursors and colonize the spleen, lymph node follicles, and other peripheral lymphoid tissues. Upon antigen exposure follicular B cells can undergo T cell dependent maturation in form of immunoglobulin class switching, hypersomatic mutation, and differentiation into plasma and memory B cells. Importantly, B2 cells are short-lived and proliferation relies upon antigen stimulation. MZ B cells are innate-like lymphocytes essentially producing natural antibodies in the absence of antigen stimulation and setting up rapid T cell-independent antibody responses against pathogenic antigens. In addition, they are involved in antigen trapping, transport, and presentation (22). In contrast to B2 cells, MZ B cells do not circulate but reside near the marginal sinus of the spleen (23).

A functional distinct B cell population develops earlier in life from hematopoietic stem cells present in the fetal liver and maintain their numbers by self-replenishment (24). As they develop earlier than B2 cells during ontogeny, this population inherited the term B1 cell. With regards to tissue location B1 cells have been found to occupy different areas when comparing human and mouse tissues. In the murine system, B1 cells pre-dominantly localize to pleural and peritoneal spaces whereas in human adults they primarily populate the peripheral blood (13, 25, 26). B1 cells have been readily identified in murine studies and are distinguished by their expression of several surface markers CD45 (B220^low^), IgM^hi^, CD23^−^, CD43^+^, and IgD^low^ that are not expressed by B2 cells (27, 28). B1 cells can be further subdivided into B-1a and B-1b cells based on distinct phenotypic features within this group. By consensus B-1 cells expressing CD5 are known as B-1a cells and those lacking the expression of CD5 are known as B-1b cells (21, 29, 30). B1 cells are part of the innate immune response and produce the majority of natural antibodies, in particular IgM against a broad spectrum of infections (31–34). These cells do not develop into memory B cells.

The most recently described subset of B2 cells is that of regulatory B cells. This unique population has been found to inhibit excessive inflammatory responses that contribute to the development of autoimmune disease. The main hallmark of regulatory B cells is the production of IL-10, a potent anti-inflammatory cytokine with pleiotropic immunoregulatory activities. Based on their secretory function, they have been labeled B10 cells in the mice and Breg in humans. However, this population exercises their function through more than one mechanism for example the secretion of TGF-β. They represent between 1 and 3% of splenic B cells but much controversy exists regarding surface marker expression. It is fair to say that B10 do have a unique phenotype but within this group are phenotypically distinct subsets. A further regulatory B cell subset with a CD1d^hi^CD5^+^ phenotype had been identified to secrete IL-10 and control T cell-dependent inflammatory responses (16). However, CD1d^hi^ is expressed by various hemopoietic-derived cells (35).

## Antibody Production

As stated earlier B2 cells are central players of humoral immunity by giving rise to differentiated antibody-secreting plasma cells. The antibody immune response is highly complex but as a simple outline once secreted, selectively produced antibodies recognize and bind particular external antigens and aid their destruction. For all that, there is a population of antibodies unable to form antigen–antibody complexes due to a structural anomaly in form of an oligosaccharide residue. These so-called asymmetric antibodies compete with their precipitating counterpart by binding the same antigen but are unable to activate effector immune mechanisms, such as complement fixation and phagocytosis. Instead, they have been speculated to function as blocking antibodies and thus may provide protection to the antigen. This blocking property has been demonstrated in previous studies (36).

Chiefly antibodies can be divided into five different classes based on their structural variability, target specificity, and distribution. *Per se* all isotopes are categorized according to their differences in the amino acid sequence in the constant region (Fc) of the heavy chain. As well, they occur in two physical forms: soluble antibodies and membrane-bound antibodies. Membrane-bound immunoglobulins form the B cell antigen receptor complex on B cells. B2 cell derived plasma cells secrete predominantly adaptive antibodies initially in form of IgM and subsequently in form of high-affinity, somatically mutated IgG. Both are dependent upon antigen stimulation. However, en masse IgM secretion is antigen-independent, which brought about the concept of two distinct types of IgM, natural IgM, and antigen-induced IgM respectively (37). Natural IgM is mainly secreted by B1 cells and to a lesser extent by MZ B cells in the complete absence of external antigenic stimulation whereas antigen-induced IgM and IgG are mostly produced by B2 cells (38–43). Antibodies from both cell types have been shown to be necessary and moreover act in concert to provide full immune protection as demonstrated by Baumgarth et al. (44).

In contrast to their adaptive counterparts natural antibodies are defined through their properties of low affinity and polyreactivity. Typically, they are able to recognize cross-reactive epitopes on encapsulated gram-positive bacteria, pathogenic viruses, apoptotic cells, and oxidized low-density lipoproteins and promote their clearance (31, 45). In this way, they provide immediate and broad protection against pathogens within the naive host, making them a crucial component of the humoral innate immune system. Unfortunately, cross reactivity of B1 and MZ B cell derived natural antibodies is not only skewed toward the recognition of pathogenic antigens but also the recognition of self-antigens provoking host cell destruction and ultimately autoimmunity. Thus, it was tempting to speculate that B1 cells may play a central role in the production autoantibodies (42, 46). However, natural antibody production is tightly regulated by the immune system and these natural antibodies rarely enter germinal centers to undergo affinity maturation. Hence, their potential for producing high-affinity antibodies with harmful specificity against their own parts is greatly restricted (45).

Surprisingly, several studies demonstrated that antibodies involved in pathogenic immune deposits within the kidneys are entirely of B2 cell origin (47). On that account, IgG antibodies have been shown to function as dominant mediators for several autoimmune diseases including systemic lupus erythematosus (SLE) and rheumatoid arthritis (RA) (48–50). The mechanisms involved in generating autoantibodies are not fully understood. However, through the process of gene segment rearrangement the immune system is capable of generating a virtually unlimited display of antibodies. Despite the establishment of multiple checkpoints which negatively select B cells with self-reactive antigen receptors, by some detrimental mechanism this genetic rearrangement may give rise to autoreactive antibodies; subsequently interacting with self-antigens and contributing toward the clinical picture of autoimmunity.

With reference to the production of natural IgM from B1 cells, there is much debate regarding their protective and destructive contribution toward autoimmune processes. Hayakawa and colleagues have demonstrated in 1999 that murine B1 cells are paradoxically positively selected for the production of autoantibodies (50). Mice deficient in serum IgM not only experienced a diminished response to pathogenic antigens. Moreover, the absence of secreted IgM stimulated the development of IgG autoantibodies (51). This was confirmed by Boes and colleagues in 2000 in normal mice unable to secrete IgM and lupus-prone lymphoproliferative (lpr) mice unable to secrete IgM. Here, lpr mice developed elevated IgG autoantibodies and experienced more severe glomerulonephritis owing to larger numbers of glomerular immune complexes (52). These and subsequent data demonstrate B1 cell-secreted IgM as a critical factor in hampering the development and severity of autoimmunity possible by means of apoptotic cell clearance (53, 54).

B1 cells have been implicated in the pathogenesis of acute inflammation and chronic autoimmune diseases in murine and human studies (55, 56). This was best witnessed in an SLE mice model in which B1 cell depletion reduced the severity of lupus autoimmune pathogenesis in (NZB × NZW) F1 mice (57). Further studies have demonstrated a significant increase of murine B1 cells as well as an increased production of self-reactive antibodies in RA and SLE (28, 58, 59). Like murine B-1a cells, human CD5^+^ B cells have been reported to produce autoantibodies in form of IgM rheumatoid factor (60). As a number of studies do support whereas others do not support the role of B1 cells involved in the pathogenesis of autoimmune disease, this area remains controversial.

## Immune Regulatory Function of B Cells in Autoimmunity, Cancer, and Transplantation

The role of B cells in the pathogenesis of autoimmune diseases extends beyond the production of autoantibodies. Rather B cells are now well-recognized for their positive and negative regulatory functions during immune responses. Newly described so-called regulatory B cells possess the ability of negatively regulating cellular immune responses and inflammation. A variety of cytokines produced by regulatory B cell subsets have been reported, with IL-10 being the most studied. The regulatory immune function was first reported by Janeway and colleagues in 1996 in a B cell deficient mice model of acute experimental autoimmune encephalomyelitis (EAE) (61). Genetically B cell-deficient mice (IL-10^−/−^) developed a persistent pro-inflammatory immune response and increased severity of EAE in comparison to wild type mice (62). Although this particular B cell regulatory effect was not IL-10 dependent, various mouse models have reinforced the importance of B cell derived IL-10 in EAE and other human autoimmune disease (62, 63). As such B10 cells have been shown to suppress the progression of intestinal inflammation in inflammatory bowel disease (IBD) and prevent the development of collagen-induced arthritis in murine models (64–66).

Studies of B10 cells and human autoimmune diseases are limited and their relevance in maintaining peripheral tolerance remains unclear. One study demonstrated the presence and moreover significantly increased production of B cell derived IL-10 in untreated RA, SLE, and Sjögren’s syndrome patients compared to controls (67). A different study defined a human B cell phenotype with regulatory capacities (67). CD19^+^CD24^hi^CD38^hi^ B cells isolated from human peripheral blood and stimulated with CD40 suppressed the differentiation of T_h_1 cells. This effect was partially mediated by IL-10. In comparison, the same B cell population isolated from the peripheral blood of SLE patients responded poorly to CD40 stimulation, produced less IL-10 and in this way lost its suppressive capacity.

In both murine and human models, the regulatory effects of B cells are very likely mediated through the anti-inflammatory effects of IL-10 and the ability of B cells to interact with pathogenic T cells to reduce harmful immune responses. IL-10 effects are mediated by multiple mechanisms such as the inhibition of the pro-inflammatory cytokine TNFα production (68, 69). B10 cells suppress T_h_1 differentiation and inhibit Ag-specific CD4^+^CD25^−^ T cell proliferation (70). This key role of B cell derived IL-10 in controlling T cell mediated autoimmunity was supported in several studies (67, 71, 72). A recent murine study identified a different IL-10 independent mechanism through which B cells can regulate autoimmunity. Here, glucocorticoid-induced TNF ligand (GITR ligand) expression by B cells was required to induce the proliferation of Treg in promoting EAE recovery (63).

Although antitumor immunity is not well understood several *in vivo* experiments have shown the regulatory action of B cells in inhibiting immune response against tumors. In B cell knockout (BKO) mice, the depletion of B cells enhanced tumor clearance in Friend murine leukemia virus gag-expressing mouse EL-4 (EL-4 gag) and D5 mouse melanoma whereas tumor progression in wild type mice was uncontrollable (73). Similarly, EL-4 thymomas, MC38 colon carcinomas, and B16 melanomas in IgM^−/−^ B cell-deficient mice exhibited spontaneous tumor regression or significant delayed growth in comparison to wild type mice (74). It has been speculated that IL-10 release from B cells inhibits CD8^+^ T cell memory development and INFγ production from CD8^+^ T and natural killer (NK) cells. Both are important for the tumor immune surveillance. Thus B cells can function as regulatory cells in some tumor settings potentially through the decreased IL-10 production from B cell depleted mice.

GVHD is a pathological condition in which donor T cells from the transplanted tissue initiate an immunologic attack on the recipient’s cells. Host APCs particular DCs are crucial for the stimulation of donor T cells and hence the induction of GVHD (75). As previously stated B cells are also able to function as APCs and given the regulatory action of B10 cells in autoimmunity and cancer, their involvement in graft versus host disease has been hypothesized and explored. Rowe and colleagues demonstrated that B cell-deficient μMT mice receiving BM transplantation demonstrated higher mortality rates due to acute GVHD than wild type recipients (76). This seems to be directly linked to the ability of B cells acting as APCs in reducing the proliferation of CD4^+^ T cells as well as the production of pro-inflammatory cytokines within the donor. Moreover, they have demonstrated that the mechanism of B cells in suppressing GVHD is directly related to IL-10 as IL-10^−/−^ mice developed more severe acute GVHD than recipient mice in which B cells are wild type (76). Finally, although most B cells are eliminated by total body irradiation preceding graft insertion, IL-10 producing B cells appear to be more resistant toward irradiation regimes. A recent cohort study aimed to identify immune parameters that would discriminate tolerant kidney transplant patients from subjects receiving immunosuppression with stable allograft function (77). This study found that tolerant recipients displayed increased total B cell numbers and naive B cells in peripheral serum and had an enhanced expression of three B cell genes in comparison with recipients receiving immunosuppression. These results may also indicate a potential regulatory role for B cells in transplantation tolerance although further studies are needed to identify whether such findings represent a cause or consequence of tolerance.

## B Cells in Pregnancy

The concept of immune tolerance and the primary function of the immune system protecting against pathogens becomes a much more complex picture with view toward mammalian pregnancy. During this period of time, the maternal immune system has the double function of tolerating a semi-allogenic fetus, expressing both maternal and paternal antigens, while maintaining the fight against infection. This fine equilibrium between maternal fetal tolerance and immune activation is orchestrated by multiple cellular players. The role of B cells herein is poorly studied, especially when taking into account the many studies dedicated to T cells in pregnancy.

## Antibody Producing B Cells in Normal Pregnancy and during Pregnancy Complications

We have previously described a population of IgG-type antibodies that bind to antigens with a relative high-affinity but fail to initiate host mechanisms facilitating the destruction of foreign antigens. Since these asymmetric antibodies seem to provide protection for antigens, it has been speculated that they may play a role in the immunological aspects of protecting the fetus against maternally derived symmetric, antipaternal antibodies at the fetomaternal interface (78). Asymmetric antibodies have been identified in human and other mammalian sera (79). Moreover, their production increased considerably in maternal serum and placental tissue during normal human pregnancy whereas their absence has been associated with pregnancy failure (80–83). One mechanism postulates that these antibodies block placental antigens in order to prevent the immunological attack by maternal NK cells and cytotoxic lymphocytes. Their secretion seems to be partially hormone regulated (84). In contrast to the protective effect proposed for asymmetric antibodies, natural antibodies facilitate pregnancy complications.

Pre-eclampsia (PE) is one of the leading causes of maternal mortality and morbidity worldwide and affects between 6 and 8% of pregnancies (85). It is defined as new onset hypertension presenting after 20 weeks gestation with significant proteinuria and resolves post-delivery. Importantly, this condition not only affects the mother but presents a high risk for the fetus in form of intrauterine death, preterm delivery, and low birth weight. With regards to the pathophysiology both immunological and genetic contributory factors have been proposed in addition to preexisting maternal diseases, i.e., chronic kidney disease and autoimmune conditions such as systemic lupus erythematosis or antiphospholipid syndrome. Pre-eclampsia is an abnormality of placentation, in particular defective remodeling of maternal uterine spiral arteries precipitating high resistance uteroplacental circulation leading to insufficient placental and in this way fetal perfusion. Cells and regulatory molecules have been implicated in the immunological alterations established in the placental microenvironment of patients with pre-eclampsia. One of the main differences in pre-eclampsia is a shift toward T_h_1 responses and the production of IFN-gamma of uncertain origin (86). Another mechanism is the blockage of transmembrane receptors for two potent angiogenic substances, vascular endothelial growth factor (VEGF) and placental growth factor (PlGF). Fms-like tyrosine kinase-1 (sFlt-1) has been identified to block these receptors and was found in high concentrations in pre-eclampsia (87–90). This endothelial dysfunction was rescued by administration of exogenous VEGF and PlGF *in vitro* studies. Furthermore heme oxygenase-1 (HO-1), an anti-inflammatory enzyme, able to inhibit sFlt-1 release has been found to be decreased in women who later developed pre-eclampsia. This is supported by data from animal models where it could be shown that HO-1 deficiency is related to uterine growth restriction and development of hypertension at midpregnancy. This seems to be dependent on the number of uterine NK cells. A HO-1 metabolite, carbon monoxide, can rescue this PE-like phenotype when applied continuously during implantation and placentation at low doses (91, 92). A different mechanism suggested autoantibodies against the vascular angiotensin II receptor type 1 (AT_1_) to account for disease manifestation. In 1999, Wallukat and colleagues were first to report the presence of circulating autoantibodies (AT1-AA) against the AT_1_ receptor in patients with pre-eclampsia, suggesting their involvement in gestational hypertension (93). The current understanding of AT1-AA and their involvement in pre-eclampsia was recently reviewed by Herse and LaMarca (94). As described earlier, CD19^+^CD5^+^B-1a B cells are a major source of natural and polyreactive antibodies. Recent evidence comes from Jensen and colleagues, as they detected a dramatically increased CD19^+^CD5^+^B-1a B cell count in peripheral blood of pre-eclamptic patients in comparison to controls having normal pregnancies. The same cells were further detected in the placenta of pre-eclamptic but not normal pregnancies. This process seems to be driven by higher chorionic gonadotropin (hCG) levels present in the serum and placenta of patients with pre-eclampsia and is supported by the fact that 95% of CD19^+^CD5^+^ cells express the hCG receptor (hCGR) and expand on hCG stimulation *in vitro* cultures. Most importantly, isolated CD19^+^CD5^+^ cells produce autoantibodies against angiotensin II type 1 receptor (95).

Lately, we have learned that pre-eclamptic women exhibit components similar to various chronic inflammatory diseases, such as elevated TNF-alpha, autoantibodies, and autoimmune associated T cells and cytokines (96–99). Recent studies focused predominantly on the role of the agonistic autoantibody to the angiotensin II type 1 receptor (AT1-AA) to account for much of the pathophysiology of pre-eclampsia (100–102). Of those several demonstrated that infusion of purified rat AT1-AA into normal pregnant rats increased blood pressure, the antiangiogenic factor sFlt-1 and sEndoglin (103, 104). To further demonstrate, the important role for B cells and endogenously generated AT1-AA in mediating hypertension in response to placental ischemia, La Marca and colleagues have proposed a model of B cell depletion to suppress endogenously generated AT1-AA. Rituximab, a chimeric murine-human monoclonal antibody against the CD20 antigen located on pre-B, immature, and mature B cells, was used to induce B cell depletion in normal pregnant and reduced uterine perfusion pressure (RUPP) rats (104). RUPP rats treated with rituximab exhibited less blood pressure increases in response to induced placental ischemia (105). This data provides potential insight into adverse pregnancy outcomes in humans and can provide a new approach to the treatment of pregnancy failure. As such a recent case report has described a successful pregnancy after rituximab treatment in a patient with a history of *in vitro* fertilization (IVF) failures and positive anti-cardiolipin antibody (ACA). Following a course of rituximab, the patient’s ACA became negative and she successfully conceived with IVF treatment (106). Rituximab destroys B cells that have CD20 on their surfaces, normal, and malignant respectively. CD20 is not expressed on stem cells and most plasma cells, thus B cell regeneration is possible and immunoglobulin synthesis by plasma cells is not affected after treatment with anti-CD20 antibody (107, 108). It has been used to treat diseases, which are characterized by excessive numbers of B cells, overactive B cells, or dysfunctional B cells. This includes several autoimmune diseases, B cell malignancies, and transplant rejection (109).

## B10/Breg Cells in Pregnancy

As the fetus is semi-allogeneic to its mother, it is reasonable to assume that the maternal immune response is a key determinant in pregnancy success and failure. In normal pregnancy, immunological adaptations are in place to protect the fetus from the maternal immune system. Failure in the accommodation of such mechanism could lead to sporadic and recurrent pregnancy loss. Recurrent miscarriage is an important complication of human gestation, affecting approximately 1% of the population (110). It is classically defined as a loss of three or more consecutive and clinically recognized pregnancies within the first trimester. Although not fully understood the underlying etiology of recurrent pregnancy is either embryological or maternal driven and can be divided into anatomical, genetic, endocrine, infectious, environmental, thrombophilic, and immunological factors (111). Focusing on the latter one mechanism postulated is the recognition of paternal antigens at the fetoplacental unit by the maternal immune system, resulting in a pro-inflammatory immune response and fetal rejection (112). The concept that B10/Breg cells immunomodulate inflammatory processes and participate in the maintenance of tolerance through IL-10 gave rise to the idea that they may control inflammatory processes in pregnancy and are important in the immunological adaptations leading to the survival of the fetus.

While researchers have reported a considerable expression of IL-10 in the decidua and placenta in mice and humans, IL-10 is not crucial for the completion of successful allogeneic pregnancies as demonstrated in breeding experiments of IL-10-null mutant (*Il10*^−/−^) mice (113–115). However, IL-10 serves an important role in protecting pregnancy from the adverse effects of inflammatory stress during gestation. This became evident in rodent studies using lipopolysaccharide (LPS) to induce pregnancy loss. *Il10*^−/−^ animals demonstrated high incidences of miscarriage whereas administration of equal doses of LPS did not affect pregnancies in the control group (116). Concomitantly, administration of recombinant IL-10 reversed the harmful effects of injected LPS (117). Of further significance was a report by Chaouat and colleagues utilizing CBA × DBA/2 mice. CBA × DBA/2 mating combinations express high rates of spontaneous fetal resorption accompanied by increased levels of local pro-inflammatory cytokines. This is likely to result from a local defect in the IL-10 production as IL-10 levels in placenta and decidua in pregnancies of CBA/J × DBA/2J mating combination have been shown to be exceptionally low. Exogenous administration of recombinant IL-10 significantly reduced LPS-induced fetal loss in CBA/J × DBA/2J but did not change outcomes of *Il10*^+/+^ mice (118). Conversely, anti-IL-10 antibodies increased resorption rates in this group (119). Furthermore in the remaining viable fetuses IL-10 deficiency predispose to growth restriction and a progressive decline in fetal weight in the presence of LPS. This was not observed in *Il10*^+/+^ mice treated with LPS (118).

Several mechanisms have been argued through which IL-10 may accomplish pregnancy preservation from LPS-induced pathologies. IL-10 is well known to inhibit the synthesis of pro-inflammatory cytokines specifically TNF-alpha from monocytes and macrophages *in* autoimmunity (120). Consequent cytokine profile analysis of maternal serum and gestational tissue following LPS injections showed elevated pro-inflammatory cytokine levels in *Il10*^−/−^ mice compared with tissues from *Il10*^+/+^ mice. Significantly raised levels were measured for TNF-α, IL6, IL1A, and IL12p40 with TNF-α most dramatically affected by IL-10 deficiency (118, 121). Further evaluation of TNF-α in mediating the adverse effects of IL-10 deficiency in pregnancy using etanercept, a TNF inhibitor, Robertson and colleagues demonstrated a partial reduction in fetal loss in IL-10^−/−^ mice treated with etanercept (118). Another study has reported an important association between fetal resorption in IL-10 deficient mice with a significant rise in uterine NK cell cytotoxicity and placental invasion. Pregnancies in LPS-treated IL-10 deficient mice could be rescued through depleting uNK cells, IL-10 administration, or TNF-alpha reversal. These results suggested an immune mechanism of fetal destruction by which uNK cells mediate inflammation in the absence of IL-10 whereas a regulatory cross-talk between IL-10 and uNK cells contributes toward a positive pregnancy outcome (122).

As discussed earlier, an important source of IL-10 comes from regulatory B cells. In our own studies, we have established that splenic B10 cells increased in frequency during normal murine pregnancy (NP). This B10 cell expansion was not evident in the non-pregnant and abortion prone (AP) group, both of which demonstrated comparable low levels. Similarly on measuring IL-10, we demonstrated an increase in IL-10 production in NP compared to non-pregnant mice. Again this augmentation was not observed in AP mice. Addressing the participation of B10 cells in establishing pregnancy tolerance, we further identified that the transfer of B10 cells from NP mice into AP animals on day 0 of pregnancy was sufficient to prevent fetal rejection (123). One possible mechanism of IL-10 action is through DCs, as they abundantly express the IL-10 receptor (IL-10R) and can alter the functionality of other immune cells. IL-10 inhibits the maturation process of monocyte derived DCs into efficient IL-12 secreting APCs consequently inhibiting their capacity to present antigens to T cells to activate them and induce the differentiation of naïve T cells to Th1 cells (124). This mechanism may be applicable for pregnancy as suggested by our data. We proved that IL-10 kept DCs in an immature state whereas DC maturation continued in the presence anti IL-10 antibody. Furthermore transferring B10 cells from NP into AP females was associated with decreased numbers of mature DCs and an augmentation of CD4^+^Foxp3^+^Treg. Foxp3^+^ regulatory T cells play a central role in sustaining maternal fetal immune tolerance and immature DCs are efficient inducers of Tregs in pregnancy (125). Overall the anti-inflammatory properties of B10 cells can provide a new approach to the treatment of spontaneous abortion due to immune mediated fetal rejection.

## Summary

**Figure 1:**
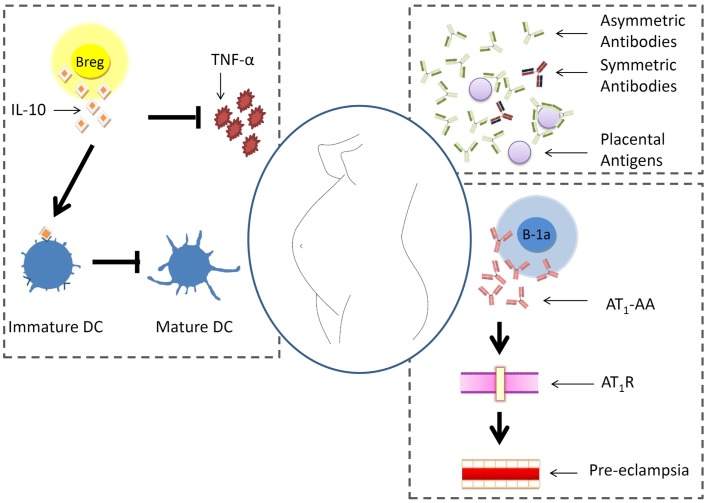
**B cell behavior during pregnancy**. Pregnancy factors promote the generation of asymmetric antibodies that protect placental antigens from immune reactions. In pre-eclampsia, B-1a B cells can turn autoreactive and secrete antibodies against the angiotensin receptor 1. In patients with autoimmune disorders, it is possible that B cells secrete more autoantibodies and endanger the gestation. A newly described B cell type, the so-called regulatory B cells, produces IL-10 and is proposed to positively influence pregnancy by hindering Th1 immune responses.

B cells are pleiotropic components of the immune system and are at the interface of innate and adaptive immunity. Their role during pregnancy is rather poorly studied. It is however known that pregnancy factors promote the generation of asymmetric antibodies that protect the fetus from immune reactions. In pre-eclampsia, B-1a B cells can turn autoreactive and secrete antibodies against the angiotensin receptor 1. In patients with autoimmune disorders, it is possible that B cells secrete more autoantibodies and endanger the gestation. A newly described B cell type, the so-called regulatory B cells, produces IL-10 and is proposed to positively influence pregnancy by hindering Th1 immune responses. This is resumed in Figure 1.

## Conflict of Interest Statement

The authors declare that the research was conducted in the absence of any commercial or financial relationships that could be construed as a potential conflict of interest.
